# Developmental Programming and Glucolipotoxicity: Insights on Beta Cell Inflammation and Diabetes

**DOI:** 10.3390/metabo10110444

**Published:** 2020-11-04

**Authors:** Marlon E. Cerf

**Affiliations:** 1 Grants, Innovation and Product Development, South African Medical Research Council, Tygerberg 7505, South Africa; marlon.cerf@mrc.ac.za; 2 Biomedical Research and Innovation Platform, South African Medical Research Council, Tygerberg 7505, South Africa; 3 Division of Medical Physiology, Department of Biomedical Sciences, Faculty of Medicine and Health Sciences, University of Stellenbosch, Tygerberg 7505, South Africa

**Keywords:** beta cell death, beta cell dysfunction, beta cell failure, ER stress, hyperglycemia, obesity, oxidative stress, saturated fatty acids

## Abstract

Stimuli or insults during critical developmental transitions induce alterations in progeny anatomy, physiology, and metabolism that may be transient, sometimes reversible, but often durable, which defines programming. Glucolipotoxicity is the combined, synergistic, deleterious effect of simultaneously elevated glucose (chronic hyperglycemia) and saturated fatty acids (derived from high-fat diet overconsumption and subsequent metabolism) that are harmful to organs, micro-organs, and cells. Glucolipotoxicity induces beta cell death, dysfunction, and failure through endoplasmic reticulum and oxidative stress and inflammation. In beta cells, the misfolding of pro/insulin proteins beyond the cellular threshold triggers the unfolded protein response and endoplasmic reticulum stress. Consequentially there is incomplete and inadequate pro/insulin biosynthesis and impaired insulin secretion. Cellular stress triggers cellular inflammation, where immune cells migrate to, infiltrate, and amplify in beta cells, leading to beta cell inflammation. Endoplasmic reticulum stress reciprocally induces beta cell inflammation, whereas beta cell inflammation can self-activate and further exacerbate its inflammation. These metabolic sequelae reflect the vicious cycle of beta cell stress and inflammation in the pathophysiology of diabetes.

## 1. Introduction

Beta cells are dynamic and respond to fluctuating demands for insulin. Inflammation contributes to the pathogenesis and is an underlying mechanism of several metabolic diseases. Developmental programming (hereafter programming) through a nutritional insult, such as maintenance on a high-fat diet (HFD) during fetal and early neonatal life, alters growth and developmental trajectories at the organ (e.g., pancreas), micro-organ (e.g., islets), and cellular (e.g., beta cell) levels that trigger the pathogenesis of metabolic diseases. In beta cells, high-fat programming (i.e., maintenance on a diet of ≥40% mainly saturated fat as energy during critical developmental windows) induces beta cell hypoplasia and hypotrophy (altered beta cell structure) that diminishes beta cell function (altered beta cell physiology) evident by impaired glucose-stimulated insulin secretion (GSIS) resulting in insufficient insulin release that results in and exacerbates hyperglycemia, as demonstrated in neonatal, weanling, and adult progeny [1,2,3,4,5,6,7,8,9]. High-fat programming also induces an altered metabolism of the substrates. For example, non-esterified fatty acids (NEFA) derived from fat metabolism have different profiles in circulation and organs that are dependent on the timing and duration of programming [10,11]. Chronic hyperglycemia contributes to further deterioration of beta cell function and to worsening insulin resistance, reflecting glucotoxicity. Chronic exposure to elevated circulating saturated fatty acids through high-fat programming induces lipotoxicity that similarly contributes to diminishing beta cell integrity and physiology and insulin resistance. Gluco- and lipo-toxicity typically co-exist as glucolipotoxicity. This article describes programming, glucolipotoxicity, and islet inflammation that precede and provoke beta cell inflammation and discusses their impact on beta cell physiology and dysfunction in the pathogenesis of diabetes.

## 2. Islet Inflammation

Macrophages are integral for inducing islet inflammation. In diabetes, intra-islet macrophage hyperplasia is the primary source of intra-islet proinflammatory cytokines [12]. Proinflammatory M1 macrophages produce and secrete interleukin 1 beta (IL1β), interleukin 6 (IL6), and tumor necrosis factor alpha (TNFα) to trigger inflammation [13], with IL1β central in islet inflammation, initiation and amplification [12]. In islets (in vitro and in vivo), M1 macrophages are the source of IL1β [12,14] and modulate beta cells’ adaptive (i.e., compensatory) response to impaired function [15], characterized by beta cell dysfunction and failure. Physiologically, resident macrophages and cytokines maintain homeostasis in beta cell development and function [16]. However, metabolic diseases are often associated with chronic systemic inflammation [16] with islet and subsequent beta cell inflammation intrinsically linked to diabetes. In islet inflammation (insulitis), proinflammatory macrophage hyperplasia concomitant with elevated cytokine and chemokine concentrations contribute to impaired islet and beta cell function [16].

## 3. Programming and Islet Inflammation

Programming refers to a stimulus or insult during critical developmental transitions that induces alterations in offspring anatomy, physiology, and metabolism that may be transient or durable, and sometimes reversible. Nutrition, through a HFD, is one way to initiate programming. Pregnant HFD-fed C57/BL6J mice were obese with increased adiposity but not overtly diabetic [17]. However, a maternal HFD during gestation and lactation induced hepatic steatosis, adipose tissue inflammation, insulin resistance, and glucose intolerance [17]. In the islets of male progeny, there was increased oxidative stress concomitant with insulin resistance and worsening beta cell dysfunction after maintenance on a HFD from conception to weaning [17]. In pancreata from juvenile Japanese macaques (Macaca fuscata) maintained on a HFD during fetal life until to 13 months of age (high fat programmed primates), there was an increase in IL6 gene expression that correlated with a blunted first-phase insulin response reflecting early beta cell dysfunction [18]. In male progeny, there was increased pancreatic IL1β gene expression and fasting glucose concentrations [18]. Furthermore, in the juvenile primate pancreas, there was islet-associated macrophage hyperplasia concomitant with an increase in proinflammatory mediators that demonstrated that innate immune infiltration occurs prior to overt obesity or glucose dysregulation [18]. These metabolic derangements manifested prior to glucose dysregulation, revealing early events in the pathogenesis of diabetes.

In non-obese diabetic mice exposed to hyperglycemia in utero, the protective compensatory factor in response to islet stress, regenerating islet-derived protein 3 gamma (Reg3g), was decreased with a high fold change [19,20] that was deleterious for postnatal islet formation and/or maturation, thereby diminishing islet cell viability and function [21]. Furthermore, many upregulated genes were associated with pathways of inflammation and cell death [21]. As systemic inflammation was absent in progeny, the enhanced inflammation did not primarily induce islet dysfunction [21]. However, the increase in the inflammatory pathway enriched transcriptome in progeny exposed to hyperglycemia during late gestation was stimulated by greater islet cell susceptibility to death [21]. Programming with hyperglycemia therefore prompts beta cell stress, death, and inflammation. Thus, beta cell inflammation is a strong inducer of beta cell death, dysfunction, and failure, and is a predictor for developing diabetes.

Obesity is associated with immune cell hyperplasia [22,23,24,25,26,27,28] and inflammation. In humans and rodents with obese pregnancies, various cytokines and chemokines were elevated viz. IL1β, IL6, IL10, TNF, interferon gamma (IFNγ) and monocyte chemoattractant protein 1 (MCP1/CCL2) [29,30,31,32], and obesity-associated maternal cytokines likely access the fetus via the placenta [33,34]. Further, maternal inflammation can initiate placental inflammation [35,36,37,38]; thus, the placenta is integral for conferring maternal obesity pathology to the fetus [39]. Therefore, obesity especially during pregnancy (maternal obesity) presents a major risk for progeny, as undesirable metabolic sequelae associated with obesity such as inflammation and hyperglycemia are conferred to progeny by their mothers.

This snapshot of the influence of programming through a HFD, hyperglycemia, and maternal obesity (often due to progeny exposed to a glucolipotoxic milieu during critical developmental phases) reveals the metabolic derangements that programming confers to progeny, e.g., hepatic steatosis, inflammation (adipose tissue, placenta and islets, and immune cell hyperplasia), insulin resistance, glucose intolerance, oxidative stress, and diminished islet and beta cell viability and function.

## 4. Glucolipotoxicity

### 4.1. Overview

Glucolipotoxicity is the combined deleterious consequences of elevated chronic glucose and saturated fatty acids (e.g., palmitic acid) concentrations on specific organs (e.g., the pancreas), and micro-organs (e.g., islets) and cells (e.g., beta cells) [40]. Glucolipotoxic-inducing diets with elevated glucose and saturated fatty acids prompt a persistent insulin demand, an increase in IAPP synthesis, potential increases in bacterial gut antigens, an increase in cytokine and chemokine production, and intra-islet inflammation that ultimately induces hyperglycemia and beta cell dysfunction [39]. With co-existing elevated lipidemia and glycemia, glucolipotoxicity manifests, resulting in metabolic alterations that drive the onset of diabetes [41,42,43].

However, during high-fat programming, glucolipotoxic effects can manifest earlier, given the particularly vulnerable life stage that the progeny are exposed to the insult. Hence, glucolipotoxicity induces derangements in beta cell structure and function, thereby inducing beta cell dysfunction; insulin resistance often follows as a consequence but can also contribute to beta cell dysfunction, and with progression to beta cell failure, diabetes ultimately manifests.

Beta cells initiate insulin transcription for pro/insulin biosynthesis and insulin exocytosis to restore glucose homeostasis, typically after ingesting a meal. Whereas glucose stimulates insulin transcription, pre-mRNA splicing and mRNA stability, proinsulin translation, maturation (to insulin) and insulin secretion, lipotoxicity and glucolipotoxicity impair several of these steps [44] and are associated with mitochondrial dysfunction [43,45]. However, persistent glucose excess, i.e., glucotoxicity, will also impair insulin processing and secretion. Ultimately, glucolipotoxicity induces beta cell dysfunction. Beta cell dysfunction, in relation to insulin secretion, presents as hyperinsulinemia during the adaptive or early pathogenesis of diabetes but evolves to hypoinsulinemia towards beta cell exhaustion and failure in the progression to overt diabetes.

Glucotoxicity is a key regulator of beta cells, whereas lipotoxicity and glucolipotoxicity were recently suggested to be less clinically relevant [46]. Elevated NEFA was acknowledged to be involved in diabetes by impairing insulin action, and despite higher NEFA concentrations in obese and diabetic individuals, there was reportedly no direct clinical evidence for beta cell lipotoxicity [46]. A supporting perspective proposed nutrient-induced metabolic stress as more relevant since NEFA are hard to trace in humans (in vivo), and therefore nutritional stress was suggested as more representative than glucolipotoxicity [47]. In a rat insulinoma beta cell line, treatment with palmitate resulted in ER expansion [48]. In db/db mouse islets and INS-1 cells treated with palmitic acid (to induce lipotoxicity), stimulator of interferon genes (STING), phosphorylated interferon regulatory factor 3 (IRF3), and IFNβ were upregulated, demonstrating that the STING-IRF3 pathway induces beta cell inflammation and apoptosis that contribute to beta cell dysfunction [49]. In another study, in human islets treated with palmitate and conducted in INS-1E cells using RNA sequencing and proteomics, there were 85 upregulated and 122 downregulated factors at the mRNA and protein levels implicated in oxidative stress, lipid metabolism, amino acid metabolism, and cell cycle pathways [50]. Lipotoxicity also affected several transcription factors implicated in metabolic and oxidative stress viz. liver X receptors (LXR), peroxisome proliferator-activated receptor alpha (PPARα), forkhead box protein O1 (FOXO1), and BTB and CNC homology 1, basic leucine zipper transcription factor 1 (BACH1) [50]. Different species and strains in animal models and varying experimental conditions in vitro and ex vivo have provided evidence for gluco-, lipo-, and glucolipotoxicity. For lipo- and glucolipotoxicity, the translational aspect to demonstrate clinical evidence and relevance remains a challenge. However, lipotoxicity and glucolipotoxicity remain relevant for beta cells, even potentially through indirect effects and under specific conditions, and hopefully, clinical studies will provide further evidence and demonstrate more direct lipotoxic and glucolipotoxic effects on beta cells to support their clinical relevance.

### 4.2. Islet and Beta Cell Glucolipotoxicity, Stress and Inflammation

Perpetual hyperglycemia and chronic saturated fatty acid exposure present as glucolipotoxicity, which contributes to islet and beta cell stress (endoplasmic reticulum (ER) and oxidative stress) and inflammation. Hyperglycemia and hyperlipidemia are traits of obesity and diabetes, and in diabetes, chronically high circulating NEFA concentrations, particularly saturated fatty acids, mediate a progressive decline in beta cell function leading to death, dysfunction [51] and failure.

In murine and human islets and beta cells, palmitate has been extensively used for the induction of ER and oxidative stress [41,52,53,54], whereas in rat islets, ex vivo exposure to elevated glucose, i.e., hyperglycemia, activated the unfolded protein response (UPR) [55]. This reflects the entwining of glucolipotoxicity, islet and beta cell stress. The exposure to high NEFA prompts superoxide (O_2_K, an anion) and peroxynitrite (ONOOK) production by beta cell mitochondria and drives NOS_2_ expression, which leads to nitric oxide (NO) production and induces ER and oxidative stress [51]. Palmitate co-cultured with healthy islets induced the release of the proinflammatory mediators (i.e., the cytokines IL6 and IL8, and the chemokine, chemokine (C-X-C motif) ligand 1 (CXCL1)) [53,56,57] with other proinflammatory cytokines viz. IL1β and TNFα also exhibited increased expression after co-culturing with palmitate [53]. Stearate and oleate (a monounsaturated fatty acid) also contribute to increased cytokine and chemokine expression [57], but inconsistently [53]. In islets, saturated fatty acids can induce chemokine (viz. Cxcl1 and Ccl3) gene expression [53,56] and initiate a microinflammatory response in the islets through the autocrine or paracrine effects of ILβ [12].

In isolated islets, the adverse effects of NEFA on GSIS are most profound with long-chain saturated fatty acids, e.g., palmitic acid [58]. Palmitic acid induces ER and oxidative stress, ceramide production, and jun *N*-terminal kinase (JNK) activation; all lead to inflammation [58,59]. In human islets, palmitic acid induced IL1β, IL6, IL8, TNFα, and chemokine (CCL2 and CXCL1) production, and also activated nuclear factor kappa-light-chain-enhancer of activated B cells (NFκB) [53]. These events demonstrate lipotoxic induced cellular stress and the provocation of inflammation in islets. Lipotoxicity and glucotoxicity can independently elicit their deleterious effects on islets and beta cells, e.g., inducing cellular stress and inflammation, also worsening diabetes [52,60]. However, they often converge as glucolipotoxicity, e.g., in obese and diabetic individuals with hyperglycemia and hyperlipidemia, which accelerates beta cell disintegration evident by cell death, dysfunction, and failure [42]. In murine islets, ethyl-palmitate infusion induced CCL2 and CXCL10 production through the toll-like receptor 4 (TLR4)-Myd88 pathway to facilitate M1 macrophage recruitment [61]. This establishes lipotoxicity as an aggravator of islet inflammation. In human diabetic pancreata and rodent diabetes, beta cells generated IL1β, i.e., were a source of hyperglycemic induced IL1β, and chronic hyperglycemia promoted intra-islet production of IL1β [62,63], which links glucotoxicity to islet inflammation. Further, IL1β alone, or IL1β with TNFα and IFNγ, prompts beta cell death, dysfunction [64,65,66], and failure with elevated glucose and NEFA concentrations, i.e., glucolipotoxicity, promoting the intra-islet production of IL1β [62,63]. Gluco-, lipo-, and glucolipotoxicity, therefore, induce islet and subsequently beta cell inflammation.

Glucolipotoxicity links beta cell stress to inflammation. Glucolipotoxicity alters mitochondrial function by impairing electron transport chain activity, which leads to increased reactive oxygen species (ROS) generation which, in turn, induces inflammation in beta cells and peripheral organs [13]. Mitochondria are close to the site of ROS generation and are therefore prone to oxidative stress [67]. The various signaling pathways that regulate mitochondrial ROS generation are involved in inflammation, ER and oxidative stress, mitochondrial biogenesis, energy demand, immune responses, and autophagy [68,69]. NEFA induce mtDNA damage dose-dependently and contribute to apoptosis [70]. Glucolipotoxicity diminishes beta cell mitochondria, inducing their fragmentation and rendering them incapable of fusion [71]. Sprague-Dawley rats maintained on a fetal and lactational HFD were insulin resistant with beta cell dysfunction (with enlarged insulin secretory granules) subsequent to reduced mtDNA content and altered mitochondrial gene expression [9]. Nutrient overload, such as supraphysiological glucose and NEFA concentrations, promotes ER stress, which impairs beta cells’ secretory efficiency, and augments inflammation and oxidative stress. This further increases circulating glucose, i.e., hyperglycemia worsens and perpetuates [13].

## 5. ER and Oxidative Stress, Inflammation and Beta Cell Dysfunction

### 5.1. ER Stress

Cellular stress induced by ER and oxidative stress are integral to islet and beta cell inflammation, death, dysfunction, and failure. Beta cells are highly specialized for insulin biosynthesis, with pro/insulin accounting for 30–50% of total protein in beta cells [59]. ER stress is a major initiator of beta cell dysfunction. Physiologically, ~20% of proinsulin misfolds in beta cells, which is ~200,000 misfolded proinsulin molecules per minute [72]. Some ER protein misfolding is expected, with misfolding multiplying proportionally to protein complexity. However, ER stress manifests only when protein misfolding exceeds a threshold, which triggers the UPR to restore ER homeostasis [51]. Therefore, to maintain beta cell integrity and physiology, it is critical to ensure that pro/insulin misfolding does not exceed the threshold.

Beta cells often encounter ER overload due to increased metabolic demand, e.g., due to rapid and increased insulin biosynthesis and secretion in response to hyperglycemia [13] to maintain beta cell physiology. Healthy beta cells respond by increasing insulin biosynthesis >10 fold compared to un-/non-stimulated beta cells [73]. However, this may exceed the beta cells’ folding capacity, thereby resulting in unfolded pro/insulin accumulation in the ER lumen, followed by ER stress [56,74] that provokes beta cell death, dysfunction, and ultimately, failure. ER stress can induce B cell lymphocyte (B cell) cytokine and chemokine gene expression [51] to initiate inflammation.

### 5.2. Oxidative Stress

The highly metabolically active beta cells are susceptible to oxidative stress due to low levels of some antioxidants, e.g., glutathione peroxidase (GPx) and catalase [75,76]. However, beta cells have other antioxidants, e.g., glutaredoxin and thioredoxin [75]. In beta cells, oxidative stress manifests due to increased ROS/reactive nitrogen species (RNS) production or the inefficiency of antioxidants to neutralize the increasing ROS/RNS levels [13].

In beta cells, ROS and RNS include free radicals, e.g., NO, O_2_K and the hydroxyl radical (OH); non-radicals, e.g., hydrogen peroxide (H_2_O_2_); or anions, e.g., ONOOK [75,77,78]. Oxidative and ER stress induce inflammation due to prolonged UPR signaling with ROS/RNS activating NFκB and the inflammasome which drives B cell and IL1β secretion [79].

The close association of oxidative and ER stress is evident as oxidative stress induces protein misfolding through the disruption of the ER redox state and disulphide bond formation, and protein misfolding prompts ROS production [78]. The detrimental combination of beta cell dysfunction, induced by oxidative and ER stress, islet inflammation, beta cell death (by apoptosis) and impaired secretory pathway function, i.e., impaired GSIS, can therefore lead to the onset of diabetes [74,80,81].

### 5.3. Beta Cell Stress and Inflammation

Several mechanisms induce ER and oxidative stress that lead to beta cell inflammation, i.e., inflamed beta cells, and beta cell death, that then activates islet-resident cells, e.g., macrophages entwined with B, T, endothelial and dendritic cells, and myofibroblasts, concomitant with an increase in leukocyte recruitment [82,83,84]. These mechanisms are geared toward re-establishing proteostasis by restoring ER capacity for handling protein processing, folding, and trafficking [13]; but should proteostasis not be restored, beta cell death ensues [85].

In beta cells, IL1β (and other proinflammatory cytokines) induces ER stress and UPR activation, and promotes NFκB-dependent inducible nitric oxide synthase (iNOS) expression and NO production [13]. Cytokine and ER stress-induced beta cell death is associated as NO production induces ER calcium depletion and stress [86]. Oxidative stress contributes to ER stress and diminishes beta cell function such as impairing insulin transcription [87] and GSIS. In human islets, ER stress (via the RNA-dependent protein kinase (PKR)-like ER kinase (PERK) and inositol-requiring enzyme 1 (IRE1) pathways) induces thioredoxin-interacting protein (TXNIP), which in turn activates the nucleotide-binding domain leucine-rich repeat (NLR) and pyrin domain containing receptor 3 (NLRP3) inflammasome and IL1β production; thus, there is bidirectional ER stress and inflammation crosstalk [88]. Further, ER stress causes and is induced by chronic beta cell inflammation (i.e., both metabolic states cause and are caused by the other) due to their interconnectedness [13]. An increase in insulin biosynthesis (beyond the threshold) promotes ER overload, the UPR and ER stress, with persistent ER stress prompting apoptosis and IL1β release (through inflammasome activation) and local cytokines inducing NFκB activation that modulates the proapoptotic gene expression [13] which would lead to beta cell death. In beta cell dysfunction, intertwined oxidative and ER stress directly influences insulin biosynthesis, with ER stress-induced UPR [51] contributing to beta cell dysfunction and failure. This reflects the interrelations of ER and oxidative stress and inflammation; each process reciprocally contributes to and exacerbates the other processes.

## 6. Glucolipotoxicity: Interrelating Islet and Beta Cell Stress, Inflammation to Dysfunction

The islet cell production of proinflammatory mediators results in ER stress, oxidative stress, mitochondrial dysfunction, and islet cell dysfunction. Overnutrition results in obesity and increased adipose tissue mass, and with perpetual nutritional glucose and saturated fatty acid overload, leads to ER stress. In humans, beta cell dedifferentiation in response to chronic overnutrition may be reversible within the first decade of diabetes [89]. Weight loss, through dietary intervention, restored the first-phase of insulin secretion, which was linked to reduced intrapancreatic triglyceride content [89]. ER stress, which results in the accumulation of misfolded pro/insulin, in turn, induces and increases beta cell inflammation that contributes to beta cell dysfunction. Some beta cell function is maintained but is compromised, and with time may lead to overt beta cell failure, where beta cells are non- or poorly functional. Beta cell dedifferentiation contributes to a reduced beta cell mass that would constrain functioning. Beta cell death is another consequence of ER and oxidative stress, and islet and beta cell inflammation.

Beta cell dysfunction initially presents in prediabetes (e.g., in obesity and insulin resistance) through their hypersecretion of insulin in response to elevated glucose concentrations. In obesity, hyperinsulinemia occurs due to the hypersecretion of insulin and compensatory response to insulin resistance [90]. In adolescents and adults, beta cell hypersecretion of insulin, in the absence of insulin resistance, was later linked to impaired glucose tolerance and diabetes [91]. This reflects the complexity of beta cell dysfunction and the progression of diabetes. Further, it provides clinical evidence that hyperinsulinemia may not be sustainable and eventually leads to the onset of diabetes as beta cells reach exhaustion. Individuals with diabetes risk factors such as familial diabetes and sedentary lifestyles had increased fat mass and dyslipidemia [91] and a worse prognosis.

In obesity and diabetes, there is an increase in circulating NEFA and ectopic fat accumulation in organs (e.g., in the liver and muscles due to adipose tissue capacitance being exceeded). In human islets treated with palmitate, there was increased islet triglyceride content concomitant with impaired function [92]. With chronic HFD feeding, there is an increase in systemic inflammation, and in IL6 and IL10 concentrations in the adipose tissue, resulting in an increase in adipose tissue inflammation. Adipose tissue insulin resistance, which is inflamed, induces modulated adipocyte sensory nerve secretion of human factors. This contributes to beta cell failure. These present inflammatory events, peripheral to the islet, along with other organ-specific inflammation, such as inflammation in the liver, muscle, heart, and kidneys that are induced by different diseases, but may be exacerbated by glucolipotoxicity. These prevailing inflammatory states, typical in obesity, co- and multi-morbidities, also interplay with islet inflammation. The interrelations of islet inflammation with systemic and organ-specific inflammation require further investigation.

**Figure 1:**
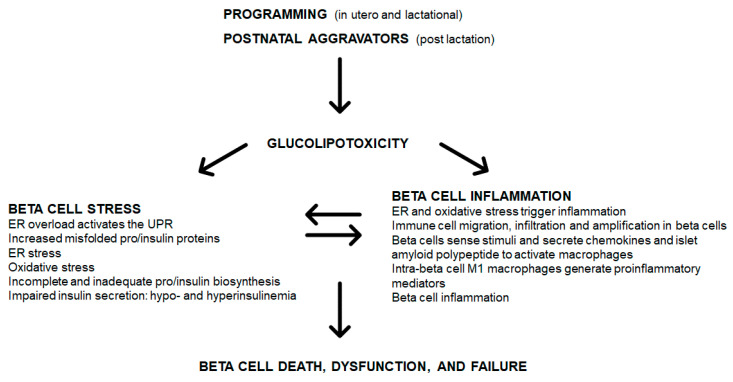
Programming, glucolipotoxicity, beta cell stress, and inflammation: convergence on beta cell death, dysfunction, and failure.

Various programming insults such as fetal and lactational exposure to a high saturated fat diet, hyperglycemia or obesity (maternal obesity), can initiate glucolipotoxicity (Figure 1). Postnatal (post-lactational) exposure to these aggravators albeit less severe, when chronic (e.g., insulin resistance and glucose intolerance reflecting hyperglycemia, and long-term or severe obesity) also induce a glucolipotoxic milieu. These only represent some triggers of glucolipotoxicity due to excess glucose and NEFA that can be derived nutritionally (e.g., hyperlipidemia and HFD overconsumption) or by a compromised metabolic state (e.g., insulin resistance, glucose intolerance and obesity). An unfavorable early-life environment has durable effects on health and increases susceptibility to diabetes through epigenetic mechanisms [93,94]. Durable epigenetic modifications are programmed by maternal overnutrition—in C57BL6/J mice, hepatic insulin receptor substrate 2 (Irs2) and mitogen-activated protein kinase 4 (Map2k4) gene methylation occurred in progeny, thereby increasing their susceptibility to diabetes [7]. In human islets that were isolated and treated with IFNγ and IL1β, there was variable epigenetic remodeling [95] mediated by inflammatory transcription factor recruitment, DNA, looping and chromatin acetylation [96]. The upregulation of inflammatory and apoptotic factors [96] contributes to beta cell dysfunction. Glucolipotoxicity, induces beta cell death, dysfunction and failure through beta cell stress and inflammation (Figure 1).

In overextended beta cells, glucolipotoxicity contributes to pro/insulin (and other) protein misfolding and accumulation in the ER lumen, resulting in ER overload which activates the UPR leading to ER stress (Figure 1). Further, in islets and beta cells, glucolipotoxicity can trigger and increase in ROS/RNS, leading to oxidative stress. ROS/RNS may regulate cell signaling pathways such as PI3K/Akt, mitogen-activated protein kinases (MAPK), JNK, and NFκB that govern cell proliferation, survival, and inflammation [13]. With resident islet and beta cell macrophages [56,97], inflammation can induce cell death through ROS/RNS [98], which links oxidative stress to islet and beta cell inflammation. ROS/RNS likely induces beta cell inflammation, beta cell death results in reduced GSIS, thereby inducing insulin resistance [99]. ER stress is also a source of ROS/RNS [99] and ER and oxidative stress are entwined and contribute to islet and beta cell inflammation. Consequentially there is incomplete and inadequate pro/insulin biosynthesis and impaired GSIS (Figure 1). Cellular stress triggers cellular inflammation, where immune cells migrate to, infiltrate and amplify in islet and beta cells leading to inflammation (Figure 1). Beta cells sense these stimuli and secrete IAPP to activate macrophages (Figure 1). The intra-islet M1 macrophages then generate more proinflammatory mediators thereby exacerbating beta cell inflammation. Beta cell stress and inflammation reciprocally cause and exacerbate each other, and over time lead to the onset of beta cell death, dysfunction, and failure (Figure 1).

**Figure 2:**
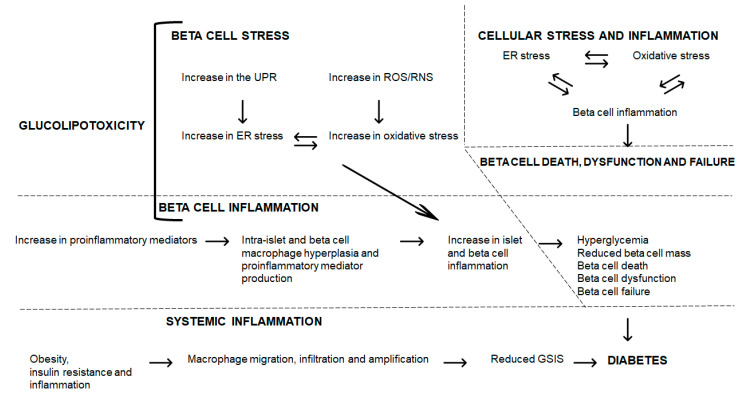
Glucolipotoxicity, beta cell stress, and inflammation: pathways to beta cell demise and diabetes.

Glucolipotoxicity induces beta cell stress with the increase in the UPR leading to ER and oxidative stress leading to increased beta cell inflammation (Figure 2). Further, glucolipotoxicity directly induces beta cell inflammation through an increase in proinflammatory mediators; within beta cells, macrophage hyperplasia and proinflammatory mediator production augment beta cell inflammation. This enhanced beta cell inflammation (through cellular stress or proinflammatory pathways) leads to hyperglycemia, reduced beta cell mass, beta cell death, dysfunction and failure, and ultimately, to diabetes (Figure 2). The entwined beta cell ER and oxidative stress and inflammation contribute to beta cell demise and failure leading to diabetes (Figure 2). These interrelations require further investigation to elucidate the mechanisms and sequence of events that lead to disease, and to identify novel agents that can counter their damaging effects on islet and beta cells. Further, systemically, obesity and inflammation can trigger macrophage migration, infiltration, and amplification, which contributes to reduced GSIS, and when prolonged, leads to diabetes (Figure 2).

Exacerbating obesity, adipose tissue insulin resistance and chronic overnutrition further prompt excess beta cell production of proinflammatory mediators. In pre-existing diabetes, islet and beta cell inflammation will exacerbate the disease and lead to further morbidities. ER stress induces and is induced by beta cell inflammation, whereas beta cell inflammation can self-activate and exacerbate beta cell inflammation. This reflects the vicious cycle of beta cell stress and inflammation in the pathophysiology of diabetes.

## 7. Conclusions

Programming, glucolipotoxicity, and the interactions of ER and oxidative stress in the pathogenesis and maintenance of disease require further unraveling and supporting evidence from clinical studies. Nutritionally and metabolically overloaded beta cells become stressed and inflamed with worsening outcomes for metabolic disease. Beta cell stress and inflammation require further investigation into adaptive mechanisms that evolve to mitigate cellular stress and inflammation, to identify strategies and targets for preserving beta cell physiology.

## References

[1] Cerf M.E., Williams K., Nkomo X.I., Muller C.J., Du Toit D.F., Louw J., Wolfe-Coote S.A. (2005). Islet cell response in the neonatal rat after exposure to a high-fat diet during pregnancy. Am. J. Physiol. Regul. Integr. Comp. Physiol..

[2] Cerf M.E., Chapman C.S., Muller C.J., Louw J. (2009). Gestational high-fat programming impairs insulin release and reduces Pdx-1 and glucokinase immunoreactivity in neonatal Wistar rats. Metabolism.

[3] Cerf M.E., Muller C.J., Du Toit D.F., Louw J., Wolfe-Coote S.A. (2006). Hyperglycaemia and reduced glucokinase expression in weanling offspring from dams maintained on a high-fat diet. Br. J. Nutr..

[4] Cerf M.E., Louw J. (2014). Islet cell response to high fat programming in neonate, weanling and adolescent Wistar rats. JOP.

[5] Gniuli D., Calcagno A., Caristo M.E., Mancuso A., Macchi V., Mingrone G., Vettor R. (2008). Effects of high-fat diet exposure during fetal life on type 2 diabetes development in the progeny. J. Lipid Res..

[6] Karbaschi R., Zardooz H., Khodagholi F., Dargahi L., Salimi M., Rashidi F. (2017). Maternal high-fat diet intensifies the metabolic response to stress in male rat offspring. Nutr. Metab..

[7] Zhang Q., Xiao X., Zheng J., Li M., Yu M., Ping F., Wang T., Wang X. (2019). A maternal high-fat diet induces DNA methylation changes that contribute to glucose intolerance in offspring. Front. Endocrinol..

[8] Zambrano E., Sosa-Larios T., Calzada L., Ibáñez C.A., Mendoza-Rodríguez C.A., Morales A., Morimoto S. (2016). Decreased basal insulin secretion from pancreatic islets of pups in a rat model of maternal obesity. J. Endocrinol..

[9] Taylor P.D., McConnell J., Khan I.Y., Holemans K., Lawrence K.M., Asare-Anane H., Persaud S.J., Jones P.M., Petrie L., Hanson M.A. (2005). Impaired glucose homeostasis and mitochondrial abnormalities in offspring of rats fed a fat-rich diet in pregnancy. Am. J. Physiol. Regul. Integr. Comp. Physiol..

[10] Cerf M.E., Louw J., Herrera E. (2015). High fat diet exposure during fetal life enhances plasma and hepatic omega-6 fatty acid profiles in fetal Wistar rats. Nutrients.

[11] Cerf M.E., Herrera E. (2016). High fat diet administration during specific periods of pregnancy alters maternal fatty acid profiles in the near-term rat. Nutrients.

[12] Eguchi K., Nagai R. (2017). Islet inflammation in type 2 diabetes and physiology. J. Clin. Investig..

[13] Keane K.N., Cruzat V.F., Carlessi R., De Bittencourt P.I.H., Newsholme P. (2015). Molecular events linking oxidative stress and inflammation to insulin resistance and β-cell dysfunction. Oxid. Med. Cell. Longev..

[14] Masters S.L., Dunne A., Subramanian S.L., Hull R.L., Tannahill G.M., Sharp F.A., Becker C., Franchi L., Yoshihara E., Chen Z. (2010). Activation of the NLRP3 inflammasome by islet amyloid polypeptide provides a mechanism for enhanced IL-1β in type 2 diabetes. Nat. Immunol..

[15] Ying W., Lee Y.S., Dong Y., Seidman J.S., Yang M., Isaac R., Seo J.B., Yang B.H., Wollam J., Riopel M. (2019). Expansion of islet-resident macrophages leads to inflammation affecting β cell proliferation and function in obesity. Cell Metab..

[16] Böni-Schnetzler M., Meier D.T. (2019). Islet inflammation in type 2 diabetes. Semin. Immunopathol..

[17] Yokomizo H., Inoguchi T., Sonoda N., Sakaki Y., Maeda Y., Inoue T., Hirata E., Takei R., Ikeda N., Fujii M. (2014). Maternal high-fat diet induces insulin resistance and deterioration of pancreatic β-cell function in adult offspring with sex differences in mice. Am. J. Physiol. Endocrinol. Metab..

[18] Nicol L.E., Grant W.F., Comstock S.M., Nguyen M.L., Smith M.S., Grove K.L., Marks D.L. (2013). Pancreatic inflammation and increased islet macrophages in insulin-resistant juvenile primates. J. Endocrinol..

[19] Marselli L., Thorne J., Dahiya S., Sgroi D.C., Sharma A., Bonner-Weir S., Marchetti P., Weir G.C. (2010). Gene expression profiles of beta-cell enriched tissue obtained by laser capture microdissection from subjects with type 2 diabetes. PLoS ONE.

[20] Xia F., Cao H., Du J., Liu X., Liu Y., Xiang M. (2016). Reg3g overexpression promotes β cell regeneration and induces immune tolerance in non-obese diabetic mouse model. J. Leukoc. Biol..

[21] Casasnovas J., Jo Y., Rao X., Xuei X., Brown M.E., Kua K.L. (2019). High glucose alters fetal rat islet transcriptome and induces progeny islet dysfunction. J. Endocrinol..

[22] Bastard J.P., Maachi M., Lagathu C., Kim M.J., Caron M., Vidal H., Capeau J., Feve B. (2006). Recent advances in the relationship between obesity, inflammation, and insulin resistance. Eur. Cytokine Netw..

[23] Khaodhiar L., Ling P.R., Blackburn G.L., Bistrian B.R. (2004). Serum levels of interleukin-6 and C-reactive protein correlate with body mass index across the broad range of obesity. J. Parenter. Enter. Nutr..

[24] Sartipy P., Loskutoff D.J. (2003). Monocyte chemoattractant protein 1 in obesity and insulin resistance. Proc. Natl. Acad. Sci. USA.

[25] Weisberg S.P., McCann D., Desai M., Rosenbaum M., Leibel R.L., Ferrante A.W. (2003). Obesity is associated with macrophage accumulation in adipose tissue. J. Clin. Investig..

[26] Das U.N. (2001). Is obesity an inflammatory condition?. Nutrition.

[27] Fried S.K., Bunkin D.A., Greenberg A.S. (1998). Omental and subcutaneous adipose tissues of obese subjects release interleukin-6: Depot difference and regulation by glucocorticoid. J. Clin. Endocrinol. Metab..

[28] Hotamisligil G.S., Shargill N.S., Spiegelman B.M. (1993). Adipose expression of tumor necrosis factor-alpha: Direct role in obesity-linked insulin resistance. Science.

[29] Catalano P.M., Presley L., Minium J., Hauguel-de Mouzon S. (2009). Fetuses of obese mothers develop insulin resistance in utero. Diabetes Care.

[30] Madan J.C., Davis J.M., Craig W.Y., Collins M., Allan W., Quinn R., Dammann O. (2009). Maternal obesity and markers of inflammation in pregnancy. Cytokine.

[31] Roberts K.A., Riley S.C., Reynolds R.M., Barr S., Evans M., Statham A., Hor K., Jabbour H.N., Norman J.E., Denison F.C. (2011). Placental structure and inflammation in pregnancies associated with obesity. Placenta.

[32] Keȩpczyńska M.A., Wargent E.T., Cawthorne M.A., Arch J.R.S., O’Dowd J.F., Stocker C.J. (2013). Circulating levels of the cytokines IL10, IFNγ and resistin in an obese mouse model of developmental programming. J. Dev. Orig. Health Dis..

[33] Zaretsky M.V., Alexander J.M., Byrd W., Bawdon R.E. (2004). Transfer of inflammatory cytokines across the placenta. Obstet. Gynecol..

[34] Dahlgren J., Samuelsson A.M., Jansson T., Holmäng A. (2006). Interleukin-6 in the maternal circulation reaches the rat fetus in mid-gestation. Pediatr. Res..

[35] Zhu M.J., Du M., Nathanielsz P.W., Ford S.P. (2010). Maternal obesity up-regulates inflammatory signaling pathways and enhances cytokine expression in the mid-gestation sheep placenta. Placenta.

[36] Challier J.C., Basu S., Bintein T., Minium J., Hotmire K., Catalano P.M., Hauguel-de Mouzon S. (2008). Obesity in pregnancy stimulates macrophage accumulation and inflammation in the placenta. Placenta.

[37] Hauguel-de Mouzon S., Guerre-Millo M. (2006). The placenta cytokine network and inflammatory signals. Placenta.

[38] Aaltonen R., Heikkinen T., Hakala K., Laine K., Alanen A. (2005). Transfer of proinflammatory cytokines across term placenta. Obstet. Gynecol..

[39] Kim D.W., Young S.L., Grattan D.R., Jasoni C.L. (2014). Obesity during pregnancy disrupts placental morphology, cell proliferation, and inflammation in a sex-specific manner across gestation in the mouse. Biol. Reprod..

[40] Poitout V., Amyot J., Semache M., Zarrouki B., Hagman D., Fontés G. (2010). Glucolipotoxicity of the pancreatic beta cell. Biochim. Biophys. Acta Mol. Cell Biol. Lipids.

[41] Cunha D.A., Hekerman P., Ladrière L., Bazarra-Castro A., Ortis F., Wakeham M.C., Moore F., Rasschaert J., Cardozo A.K., Bellomo E. (2008). Initiation and execution of lipotoxic ER stress in pancreatic β-cells. J. Cell Sci..

[42] El-Assaad W., Buteau J., Peyot M.L., Nolan C., Roduit R., Hardy S., Joly E., Dbaibo G., Rosenberg L., Prentki M. (2003). Saturated fatty acids synergize with elevated glucose to cause pancreatic β-cell death. Endocrinology.

[43] Somesh B.P., Verma M.K., Sadasivuni M.K., Mammen-Oommen A., Biswas S., Shilpa P.C., Reddy A.K., Yateesh A.N., Pallavi P.M., Nethra S. (2013). Chronic glucolipotoxic conditions in pancreatic islets impair insulin secretion due to dysregulated calcium dynamics, glucose responsiveness and mitochondrial activity. BMC Cell Biol..

[44] Lytrivi M., Castell A.L., Poitout V., Cnop M. (2020). Recent insights into mechanisms of β-cell lipo- and glucolipotoxicity in type 2 diabetes. J. Mol. Biol..

[45] Barlow J., Affourtit C. (2013). Novel insights into pancreatic β-cell glucolipotoxicity from real-time functional analysis of mitochondrial energy metabolism in INS-1E insulinoma cells. Biochem. J..

[46] Weir G.C. (2020). Glucolipotoxicity, β-cells, and diabetes: The emperor has no clothes. Diabetes.

[47] Prentki M., Peyot M.L., Masiello P., Murthy Madiraju S.R. (2020). Nutrient-induced metabolic stress, adaptation, detoxification, and toxicity in the pancreatic β-cell. Diabetes.

[48] Pinnick K., Neville M., Clark A., Fielding B. (2010). Reversibility of metabolic and morphological changes associated with chronic exposure of pancreatic islet β-cells to fatty acids. J. Cell. Biochem..

[49] Hu H.Q., Qiao J.T., Liu F.Q., Wang J.B., Sha S., He Q., Cui C., Song J., Zang N., Wang L.S. (2020). The STING-IRF3 pathway is involved in lipotoxic injury of pancreatic β cells in type 2 diabetes. Mol. Cell. Endocrinol..

[50] Lytrivi M., Ghaddar K., Lopes M., Rosengren V., Piron A., Yi X., Johansson H., Lehtiö J., Igoillo-Esteve M., Cunha D.A. (2020). Combined transcriptome and proteome profiling of the pancreatic β-cell response to palmitate unveils key pathways of β-cell lipotoxicity. BMC Genom..

[51] Hasnain S.Z., Prins J.B., McGuckin M.A. (2016). Oxidative and endoplasmic reticulum stress in β-cell dysfunction in diabetes. J. Mol. Endocrinol..

[52] Cnop M., Ladrière L., Igoillo-Esteve M., Moura R.F., Cunha D.A. (2010). Causes and cures for endoplasmic reticulum stress in lipotoxic β-cell dysfunction. Diabetes Obes. Metab..

[53] Igoillo-Esteve M., Marselli L., Cunha D.A., Ladrière L., Ortis F., Grieco F.A., Dotta F., Weir G.C., Marchetti P., Eizirik D.L. (2010). Palmitate induces a pro-inflammatory response in human pancreatic islets that mimics CCL2 expression by beta cells in type 2 diabetes. Diabetologia.

[54] Hasnain S.Z., Borg D.J., Harcourt B.E., Tong H., Sheng Y.H., Ng C.P., Das I., Wang R., Chen A.C., Loudovaris T. (2014). Glycemic control in diabetes is restored by therapeutic manipulation of cytokines that regulate beta cell stress. Nat. Med..

[55] Elouil H., Bensellam M., Guiot Y., Vander Mierde D., Pascal S.M., Schuit F.C., Jonas J.C. (2007). Acute nutrient regulation of the unfolded protein response and integrated stress response in cultured rat pancreatic islets. Diabetologia.

[56] Ehses J.A., Perren A., Eppler E., Ribaux P., Pospisilik J.A., Maor-Cahn R., Gueripel X., Ellingsgaard H., Schneider M.K., Biollaz G. (2007). Increased number of islet-associated macrophages in type 2 diabetes. Diabetes.

[57] Böni-Schnetzler M., Boller S., Debray S., Bouzakri K., Meier D.T., Prazak R., Kerr-Conte J., Pattou F., Ehses J.A., Schuit F.C. (2009). Free fatty acids induce a proinflammatory response in islets via the abundantly expressed interleukin-1 receptor I. Endocrinology.

[58] Van Raalte D.H., Diamant M. (2011). Glucolipotoxicity and beta cells in type 2 diabetes mellitus: Target for durable therapy?. Diabetes Res. Clin. Pract..

[59] Imai Y., Dobrian A.D., Morris M.A., Nadler J.L. (2013). Islet inflammation: A unifying target for diabetes treatment?. Trends Endocrinol. Metab..

[60] Donath M.Y., Gross D.J., Cerasi E., Kaiser N. (1999). Hyperglycemia-induced beta-cell apoptosis in pancreatic islets of Psammomys obesus during development of diabetes. Diabetes.

[61] Eguchi K., Manabe I., Oishi-Tanaka Y., Ohsugi M., Kono N., Ogata F., Yagi N., Ohto U., Kimoto M., Miyake K. (2012). Saturated fatty acid and TLR signaling link β cell dysfunction and islet inflammation. Cell Metab..

[62] Ehses J.A., Böni-Schnetzler M., Faulenbach M., Donath M.Y. (2008). Macrophages, cytokines and β-cell death in type 2 diabetes. Biochem. Soc. Trans..

[63] Maedler K., Sergeev P., Ris F., Oberholzer J., Joller-Jemelka H.I., Spinas G.A., Kaiser N., Halban P.A., Donath M.Y. (2002). Glucose-induced β cell production of IL-1β contributes to glucotoxicity in human pancreatic islets. J. Clin. Investig..

[64] Mandrup-Poulsen T. (1996). The role of interleukin-1 in the pathogenesis of IDDM. Diabetologia.

[65] Rabinovitch A., Suarez-Pinzon W.L., Shi Y., Morgan A.R., Bleackley R.C. (1994). DNA fragmentation is an early event in cytokine-induced islet beta-cell destruction. Diabetologia.

[66] Iwahashi H., Hanafusa T., Eguchi Y., Nakajima H., Miyagawa J., Itoh N., Tomita K., Namba M., Kuwajima M., Noguchi T. (1996). Cytokine-induced apoptotic cell death in a mouse pancreatic beta-cell line: Inhibition by Bcl-2. Diabetologia.

[67] Li N., Frigerio F., Maechler P. (2008). The sensitivity of pancreatic β-cells to mitochondrial injuries triggered by lipotoxicity and oxidative stress. Biochem. Soc. Trans..

[68] Sena L.A., Chandel N.S. (2012). Physiological roles of mitochondrial reactive oxygen species. Mol. Cell.

[69] Bolisetty S., Jaimes E.A. (2013). Mitochondria and reactive oxygen species: Physiology and pathophysiology. Int. J. Mol. Sci..

[70] Grishko V., Rachek L., Musiyenko S., LeDoux S.P., Wilson G.L. (2005). Involvement of mtDNA damage in free fatty acid-induced apoptosis. Free Radic. Biol. Med..

[71] Molina A.J., Wikstrom J.D., Stiles L., Las G., Mohamed H., Elorza A., Walzer G., Twig G., Katz S., Corkey B.E. (2009). Mitochondrial networking protects β-cells from nutrient-induced apoptosis. Diabetes.

[72] Sun J., Cui J., He Q., Chen Z., Arvan P., Liu M. (2015). Proinsulin misfolding and endoplasmic reticulum stress during the development and progression of diabetes. Mol. Asp. Med..

[73] Eizirik D.L., Cardozo A.K., Cnop M. (2008). The role for endoplasmic reticulum stress in diabetes mellitus. Endocr. Rev..

[74] Eizirik D.L., Miani M., Cardozo A.K. (2013). Signalling danger: Endoplasmic reticulum stress and the unfolded protein response in pancreatic islet inflammation. Diabetologia.

[75] Newsholme P., Rebelato E., Abdulkader F., Krause M., Carpinelli A., Curi R. (2012). Reactive oxygen and nitrogen species generation, antioxidant defenses, and β-cell function: A critical role for amino acids. J. Endocrinol..

[76] Gehrmann W., Elsner M., Lenzen S. (2010). Role of metabolically generated reactive oxygen species for lipotoxicity in pancreatic β-cells. Diabetes Obes. Metab..

[77] Lenzen S. (2008). Oxidative stress: The vulnerable β-cell. Biochem. Soc. Trans..

[78] Cao S.S., Kaufman R.J. (2014). Endoplasmic reticulum stress and oxidative stress in cell fate decision and human disease. Antioxid. Redox Signal..

[79] Menu P., Mayor A., Zhou R., Tardivel A., Ichijo H., Mori K., Tschopp J. (2012). ER stress activates the NLRP3 inflammasome via an UPR-independent pathway. Cell Death Dis..

[80] Donath M.Y., Dalmas É., Sauter N.S., Böni-Schnetzler M. (2013). Inflammation in obesity and diabetes: Islet dysfunction and therapeutic opportunity. Cell Metab..

[81] Montane J., Cadavez L., Novials A. (2014). Stress and the inflammatory process: A major cause of pancreatic cell death in type 2 diabetes. Diabetes Metab. Syndr. Obes. Targets Ther..

[82] Toyama H., Takada M., Tanaka T., Suzuki Y., Kuroda Y. (2003). Characterization of islet-infiltrating immunocytes after pancreas preservation by two-layer (UW/perfluorochemical) cold storage method. Transpl. Proc..

[83] Coppieters K.T., Dotta F., Amirian N., Campbell P.D., Kay T.W., Atkinson M.A., Roep B.O., von Herrath M.G. (2012). Demonstration of islet-autoreactive CD8 T cells in insulitic lesions from recent onset and long-term type 1 diabetes patients. J. Exp. Med..

[84] Calderon B., Carrero J.A., Ferris S.T., Sojka D.K., Moore L., Epelman S., Murphy K.M., Yokoyama W.M., Randolph G.J., Unanue E.R. (2015). The pancreas anatomy conditions the origin and properties of resident macrophages. J. Exp. Med..

[85] Xu C., Bailly-Maitre B., Reed J.C. (2005). Endoplasmic reticulum stress: Cell life and death decisions. J. Clin. Invest..

[86] Oyadomari S., Takeda K., Takiguchi M., Gotoh T., Matsumoto M., Wada I., Akira S., Araki E., Mori M. (2001). Nitric oxide-induced apoptosis in pancreatic β cells is mediated by the endoplasmic reticulum stress pathway. Proc. Natl. Acad. Sci. USA.

[87] Kaneto H., Matsuoka T.A. (2015). Role of pancreatic transcription factors in maintenance of mature β-cell function. Int. J. Mol. Sci..

[88] Oslowski C.M., Hara T., O’Sullivan-Murphy B., Kanekura K., Lu S., Hara M., Ishigaki S., Zhu L.J., Hayashi E., Hui S.T. (2012). Thioredoxin-interacting protein mediates ER stress-induced β cell death through initiation of the inflammasome. Cell Metab..

[89] White M.G., Shaw J.A., Taylor R. (2016). Type 2 diabetes: The pathologic basis of reversible β-cell dysfunction. Diabetes Care.

[90] Ferrannini E., Natali A., Bell P., Cavallo-Perin P., Lalic N., Mingrone G. (1997). Insulin resistance and hypersecretion in obesity. J. Clin. Invest..

[91] Tricò D., Natali A., Arslanian S., Mari A., Ferrannini E. (2018). Identification, pathophysiology, and clinical implications of primary insulin hypersecretion in nondiabetic adults and adolescents. JCI Insight.

[92] Lalloyer F., Vandewalle B., Percevault F., Torpier G., Kerr-Conte J., Oosterveer M., Paumelle R., Fruchart J.C., Kuipers F., Pattou F. (2006). Peroxisome proliferator-activated receptor α improves pancreatic adaptation to insulin resistance in obese mice and reduces lipotoxicity in human islets. Diabetes.

[93] Aleliunas R.E., Aljaadi A.M., Laher I., Glier M.B., Green T.J., Murphy M., Miller J.W., Devlin A.M. (2016). Folic acid supplementation of female mice, with or without vitamin B-12, before and during pregnancy and lactation programs adiposity and vascular health in adult male offspring. J. Nutr..

[94] Reusens B., Theys N., Dumortier O., Goosse K., Remacle C. (2011). Maternal malnutrition programs the endocrine pancreas in progeny. Am. J. Clin. Nutr..

[95] Peters L., Posgai A., Brusko T.M. (2019). Islet–immune interactions in type 1 diabetes: The nexus of beta cell destruction. Clin. Exp. Immunol..

[96] Ramos-Rodríguez M., Raurell-Vila H., Colli M.L., Alvelos M.I., Subirana-Granés M., Juan-Mateu J., Norris R., Turatsinze J.V., Nakayasu E.S., Webb-Robertson B.J. (2019). The impact of proinflammatory cytokines on the β-cell regulatory landscape provides insights into the genetics of type 1 diabetes. Nat. Genet..

[97] Böni-Schnetzler M., Ehses J.A., Faulenbach M., Donath M.Y. (2008). Insulitis in type 2 diabetes. Diabetes Obes. Metab..

[98] Choudhury S., Ghosh S., Gupta P., Mukherjee S., Chattopadhyay S. (2015). Inflammation-induced ROS generation causes pancreatic cell death through modulation of Nrf2/NF-κB and SAPK/JNK pathway. Free Radic. Res..

[99] Burgos-Morón E., Abad-Jiménez Z., Martinez de Marañon A., Iannantuoni F., Escribano-López I., López-Domènech S., Salom C., Jover A., Mora V., Roldan I. (2019). Relationship between oxidative stress, ER stress, and inflammation in type 2 diabetes: The battle continues. J. Clin. Med..

